# Evidence-based guiding principles to build public trust in personal data use in health systems

**DOI:** 10.1177/20552076221111947

**Published:** 2022-07-17

**Authors:** Felix Gille, Sarah Smith, Nicholas Mays

**Affiliations:** 1 27217 University of Zurich, Digital Society Initiative (DSI), Zürich, CH, Switzerland; 2 27217 University of Zurich, Institute for Implementation Science in Health Care (IfIS), Zürich, CH, Switzerland; 3 Department of Health Services Research and Policy, Faculty of Public Health and Policy, 4906 London School of Hygiene and Tropical Medicine, London, UK

**Keywords:** Public trust, trust, health system, healthcare, personal data, digital health, COVID-19, health policy, public policy, governance

## Abstract

**Objective:**

Public trust in health systems is pivotal for their effective and efficient functioning. In particular, public trust is essential for personal data use, as demonstrated in debates in many countries, for example, about whether data from COVID-19 contact tracing apps should be pooled or remain on individuals’ smartphones. Low levels of public trust pose a risk not only to health system legitimacy but can also harm population health.

**Methods:**

Synthesising our previous qualitative and theoretical research in the English National Health Service which enabled us to conceptualise the nature of public trust in health systems, we present guiding principles designed to rebuild public trust, if lost, and to maintain high levels of public trust in personal data use within the health system, if not.

**Results:**

To build public trust, health system actors need to not rush trust building; engage with the public; keep the public safe; offer autonomy to the public; plan for diverse trust relationships; recognise that trust is shaped by both emotion and rational thought; represent the public interest; and work towards realising a net benefit for the health system and the public.

**Conclusions:**

Beyond policymakers and government officials, the guiding principles address a wide range of actors within health systems so that they can work collectively to build public trust. The guiding principles can be used to inform policymaking in health and health care and to analyse the performance of different governments to see if those governments that operate in greater conformity with the guiding principles perform better.

## Introduction

Public trust is essential for the well-functioning of health systems and public participation in health care activities.^1–3^ From the viewpoint of the system, the main justification for caring about public trust is that, if there is no public trust in health system actors and their activities, these actors lack public legitimacy to act and the level of public participation in collective health system activities such as vaccination drops low.^4,5^ Public trust is embedded in principles of good governance, law and ethics which are fundamental pillars of health system activities^6–8^. Moreover, indications of levels of public trust can serve as quality indicators, be part of health policy evaluation and point towards the need for health system reforms.^9^ If public trust in health care activities is missing, potential consequences are increased transaction costs, poorer public health and, in the extreme, the entire failure of health care activities.^10–12^

Research on public trust in personal data use within the health system has gained momentum in recent years. Its importance has been further highlighted in the response to the COVID-19 pandemic.^13–15^ Public trust is particularly important for the success of a range of data-driven health system activities such as public uptake of contact tracing apps during the COVID-19 pandemic,^16–19^ public and professional acceptance of artificial intelligence applications in health care,^20–22^ the establishment of integrated electronic health data records and personal data sharing,^23–27^ donation of bio samples and personal data for research purposes^28–30^ and the public acceptance of digital health provision more broadly.^31^ Research in this area shows that public trust is a complex phenomenon that is not easy to maintain and build. Several issues contribute to this complexity. First, people can trust different actors in the same context differently. For example, we might trust nurses to tell the truth but not ministers of health, yet both are integral to the health system.^32^ Second, trust is influenced by the design of the health system. For example, the complex relationship between the public and private subsystems in Australia that offer choices about hospitals and doctors for ‘private patients’ and limited choices for ‘public patients’ influences the formation of trust in the system differently in the two groups.^33,34^ Third, scandals are often followed by public debates about whether the public trust has been damaged and how it may be rebuilt. Examples are *The Implant Files* in the *Guardian* newspaper in the UK investigating ‘faulty medical implants and their effect on patients’;^35^ the series of homicides by nurse, Victorino Chua, in 2011 in Stepping Hill Hospital Manchester, England, or by Niels Högel in Oldenburg and Delmenhorst between 1999 and 2005 in Germany.^36^ Fourth, poorly thought through initiatives involving personal data, can also harm public trust, such as the care.data initiative in England and My Health Record in Australia. Both had to be withdrawn in response to high levels of public and professional concern expressed regarding privacy, data safety and trustworthiness.^37,38^ The successor 2021 NHS Digital service in England, General Practice Data for Planning and Research, which aimed to make primary health data available to researchers and private companies, also had to be put on hold after a wave of opt outs due to privacy concerns.^39,40^ Little seemed to have been learned over time. Fifth, as the health system is interwoven with other political subsystems, spill-over effects of low levels of public trust in other political subsystems can reduce public trust in the health system.^41^ Last, aside from the health system context and political level (local, national or international), the target of the trust relationship, culture, social norms and values and demography can influence the nature of public trust.^42–45^ This contextual and cultural specificity of trust means that care must be taken when generalising across different health systems.

In media debate, the term trust can be used so loosely as to become no more than a fashionable ‘buzz word’, as, for example, in advertising by health insurance companies and in political debate where inflationary use of the term not only risks overstraining the concept but also diminishes its value for the health system.^46^ Similarly, a one-sided media focus on health care scandals, as shown in much media coverage of the UK NHS, not only undermines public trust but also supports ill-judged government policy responses.^47^ It is no secret that the present public discourse and sentiment in many countries is highly critical towards governments and public institutions, and, thereby, often scrutinises their trustworthiness.^48^ It might be, that excessive criticism of health care institutions is ultimately self-defeating because it undermines trust so much that the system deteriorates still further.

## What is public trust in the health system?

In our previous study, we developed the concept of public trust in the health system by analysing qualitative data related to three case studies drawn from the English NHS which focused on personal data use within the health system: (a) online news readership comments about the care.data programme; (b) participant interviews about experiences and perceptions of contributing to biobank research; and (c) public focus groups about perceptions of the 100,000 Genomes Project.^4^ Public trust in the health system is a relational construct existing between the public and the health system. While patient trust in health professionals contributes to the development of public trust in the system as a whole, public trust in the system as a whole is not merely the aggregated average of these micro-level trust relationships. At the individual level, trust can be defined as ‘a bet about the future contingent actions of others’.^49^ At the public level, the public trusts the health system in anticipation of a net benefit for the health system and society. Public trust develops in the public sphere by open public discourse between a range of health system actors.^50^ Examples of relevant actors are patients, healthy citizens, clinicians, politicians, media, health care industries, third sector organisations, religious bodies and health system analysts. Aside from these actors, public trust also develops based on the existence of system guarantees such as governance structures, laws and regulations or professional codes.^50^ Contemporary examples of the public sphere relevant to the health system are online fora, Twitter or other web-based social media applications, as well as more traditional mass media use and face-to-face gatherings such as community meetings.^37,51,52^

**Figure 1.**
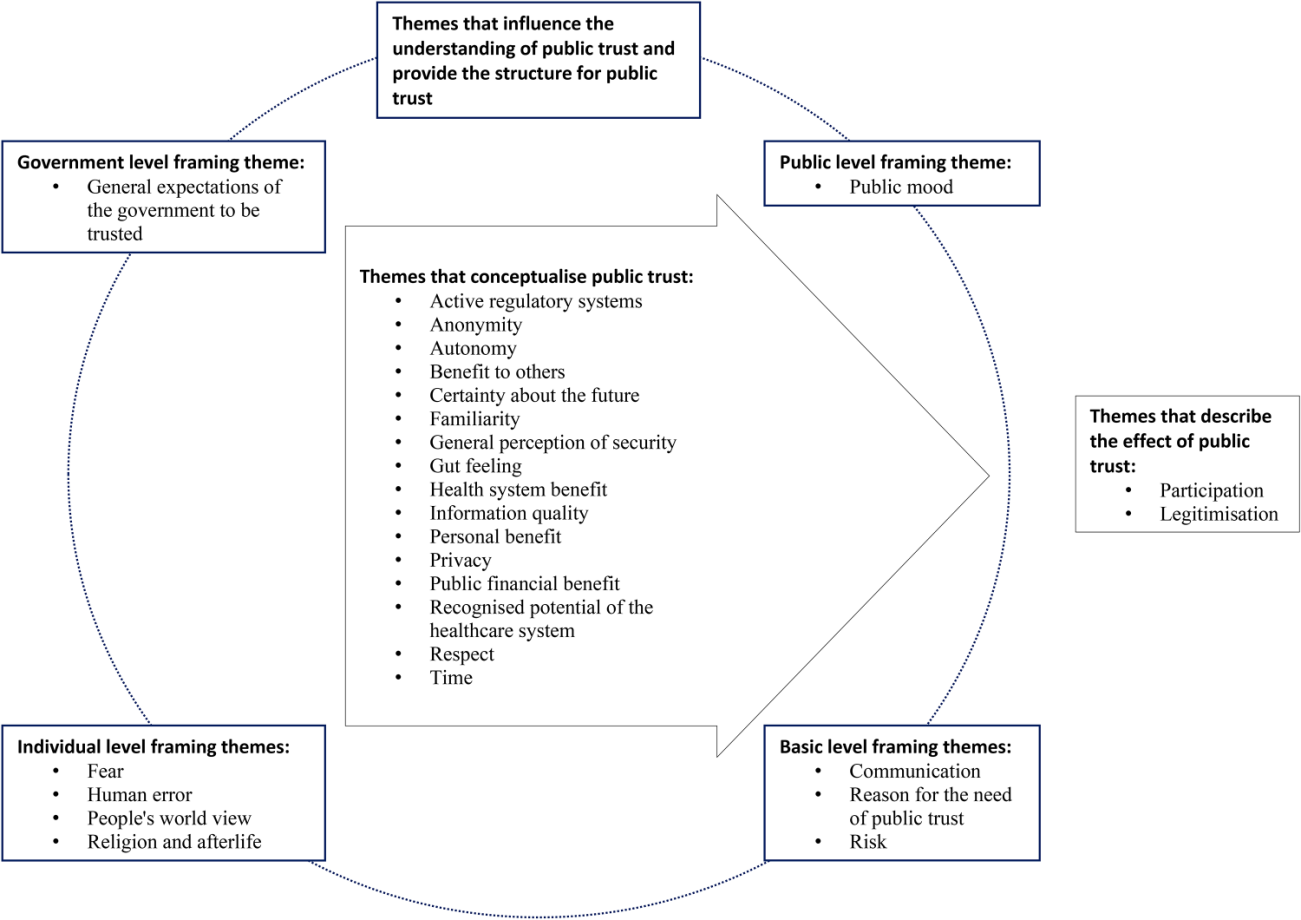
Conceptual framework of public trust in the health system.

By trusting, the public legitimises the health system and willingly takes part in health care system activities. For the public to be able to develop trust in the health system, health system actors need to provide truthful information that relates to the past, present and future. For example, when actors are communicating about a new technology, we are more inclined to trust if we have had positive experiences with a comparable existing technology. If existing medical apps keep our personal data safe, we are inclined to trust that new medical apps will do the same. We like to understand the present potential and capabilities of that technology to achieve what we might trust it for. Last, we like to understand what the technology in the near and far future will do to achieve what we might trust it for. To be trustworthy, the information we receive about an innovation needs to cover the themes that comprise the concept of public trust as shown in the arrow in Figure 1. Figure 1 summarises the conceptual model of public trust in the health system which forms the basis for the guiding principles below (for a detailed discussion, see our previous publication^4^).

To respond to this complexity and informed by our previous research to develop a conceptual framework of public trust in the health system derived from empirical work in the English NHS,^4,50^ we propose a general set of guiding principles for health policymakers and other system actors designed to maintain or increase public trust in personal data use within health systems.

## Methods

Building on the results of our previous research on public trust in the English NHS, as described in the previous section we developed the guiding principles for building and maintaining public trust iteratively^53^; where applicable, and without losing meaning, we combined several themes from the conceptual framework in Figure 1 into one guiding principle to reduce the number of guiding principles to a usable number, as shown in online Supplemental material. The iteration comprised of several cycles of revision to find the ‘sweet-spot’ where the guiding principles are specific enough to be meaningful yet general enough to be transferable across health systems and activities. To make the guiding principles tangible, we provide practical examples related to each guiding principle focused on the case of use of personal data to control the COVID-19 pandemic and other health system activities. From our experience, careful development and abstraction of guiding principles are necessary as underlying conceptions of trust are often context- and culture-specific. In line with Jaccard and Jacoby, abstraction allows us to observe commonalities between different contexts.^54^ In turn, a higher degree of abstraction demands that the guiding principles be tailored to the context in which they will be applied before they are used. With this level of abstraction, we see great value in the transferability of the guiding principles across different health systems. One guiding principle, ‘Plan for diverse trust relationships’, developed from our work describing how public trust develops in the public sphere.^50^ We added this guiding principle to account for the complexity of trust relationships in the health system.

We propose that the guiding principles are applied together and not selectively. The guiding principles are not ranked according to their importance and are presented in alphabetic order. The guiding principles could be ranked in relative importance for a specific context, yet, as the purpose of this article is to provide generally valid guiding principles, such a ranking would not be helpful. The guiding principles are aimed at health care actors in a range of different health systems as diverse as the NHS in England and the German health system. We showed in an earlier study that the development and maintenance of public trust in the health system is a community effort involving a range of different health system actors working together.^50^ Therefore, we refrained at this point to direct the guiding principles to specific actors, as depending on the context and system in which the guiding principles will be used, different actors might oversee implementation of the guiding principles. Since the guiding principles were developed on the basis of a robust conceptual framework of public trust in the health system, it seems appropriate to use them as a high-level guide for health system policymakers and leaders. They are also suitable to be used to evaluate the performance of health systems in building and retaining public trust. Although developed empirically in the universal public part of England's health care sector, we argue elsewhere that the conceptual framework is transferable to other countries with similar norms, values and understanding of what a health system should be.^4^

## How health system actors can foster public trust in personal data use in health systems

Others published guiding principles on how to foster trust in parts of the health system such as consumer trust in the food system,^55^ trust in vaccination programmes,^56^ trust in measures to fight against the 2009 H1NI pandemic^57^ or public trust in health information exchange.^58^ We present eight guiding principles that are applicable across the health system and tailored to personal data use within the health system. In summary, the guiding principles on how to foster public trust in personal data use within the health system are as follows:

- Do not rush trust building.
- Engage with the public.
- Keep the public safe.
- Offer autonomy to the public.
- Plan for diverse trust relationships.
- Recognise that trust is shaped by both emotion and rational thought.
- Represent the public interest.
- Work towards realising a net benefit for the health system and the public.

### Do not rush trust building

In contrast to the sometimes time-consuming process of building trust, once it exists, trust can be rapidly lost. This imbalance between the time necessary to build trust and the pace of destroying trust should remind us of the sensitivity and value of public trust. People need time to decide to take part in health system activities such as data donation for research or vaccination that depend on public trust. We should not expect that trust will be established quickly. The slow growth in downloads of COVID-19 tracing apps in some countries can be interpreted as showing that public trust in these apps was slow to grow.^19,59,60^ In the context of COVID-19 vaccination, Jiang argues that trust cannot and should not be rushed.^61^ Therefore, policy implementation should not be undertaken in a way that suggests haste. A realistic timeframe is key to successfully building public trust in an initiative.

### Engage with the public

Public trust evolves from free and open public debate in the public sphere. Two-way communication is the lifeblood of public trust. Communication by policy agencies needs to clearly convey the anticipated benefit of any policy proposal, to show how the policy will be implemented and to explain how the policy relates to past comparable policies or health care actions. An example of where such communication failed is the leaflet that was distributed to all households in England about the care.data programme. This leaflet did not relate the care.data programme sufficiently to past data initiatives.^62^ The information provided was also insufficient to explain the mechanisms in place to protect personal data in the new programme and there was little information on how anticipated benefits would be achieved. Furthermore, the bulk of the public consultation took place after the leaflets had been distributed, and after the programme organisers had realised that the information campaign and the programme itself was at risk of failing.^63^ These types of communication have to happen before policy implementation. Vokinger and colleagues show that in some countries COVID-19 apps were introduced without clear information about, for example, data privacy.^31^ However, providing information about privacy settings and future data use are key to building trustworthy health apps as well as enabling the donation of personal data for future research use as by biobanks.^28,64^

The information provided by policymakers must be explanatory, clear and truthful. The challenge is that people may have different perceptions of what is considered truthful, as seen in the anti-vaccination movement or COVID-19 social media debate on vaccination.^65,66^ Public information must be supported with robust scientific evidence and explanations for policy decisions must be honest about the underlying values informing those decisions. In settings where misinformation and conspiracy theories are dominant, as is the case among many members of the anti-vaccination movement, credibility and epistemic authority is of particular importance.^67^ Studies show that conspiracy believers are more mistrusting and less knowledgeable about COVID-19 or other health care activities.^68,69^ Also, belief in conspiracies influences people's willingness to disclose personal data online.^70^

The source providing information must be reliable and, when several sources agree, people trust it more. There is thus a particular problem where people consult online sources or experts disagree. Research shows that health information provided online can be of lower quality, if not misleading or wrong, compared with information provided by authoritative medical staff^71–74^. Therefore, people need to be helped to understand the variable quality of online information and need to be supported to identify poor quality information. Increased critical health literacy is associated with a lower likelihood of believing in conspiracies, of failing to spot bogus claims on the internet, and a higher likelihood of making better health-related decisions.^74^ Policymakers will need to continuously challenge and counteract bogus and misleading information provided online. When considering patients seeking advice online, policymakers should be aware of the potentially negative impact of incorrect or actively misleading information online provided by others. A solution to this problem is quality assurance processes for online sites and sources. Increased health literacy, as well as high-quality information, can contribute to trust building.^75^ With respect to health literacy and social status, research from Denmark shows that both low levels and high levels of health literacy can lead to people having lower levels of trust in public hospitals. People with low levels of health literacy and low social status can perceive the health system as not cooperative whereas people with the highest levels of health literacy and social status have expectations towards the health system that the system is unable to fulfil. These findings suggest that tailored approaches towards different groups in society are needed to increase levels of trust.^76^

The information provided should also elicit a feeling of familiarity, as familiarity with health system actors fosters trust.^77^ Similar is true for trust in health information exchange and data sharing during clinical trials.^77,78^ This observation has at least two implications for communication: (a) policymakers need to use the same style of communication to convey a range of different information and advice rather than swapping the styles depending on the topic and (b) the communication about new health system activities should entail comparable information to existing health system activities. This way the public can imagine what the new activities might look like more easily. Familiarity can play out at the interpersonal level and is most often associated with the behaviour of medical staff towards their patients. Also, familiarity with brands is considered a key factor for the trustworthiness of health apps.^64^ This suggests that COVID-19 policies should incorporate, where appropriate, publicly known partners to elicit familiarity.

All health care actors need to work together to build public trust. The responsibility to maintain and increase public trust is the responsibility of all parts of the health system. It is false to assume that (a) responsibility can be passed to others in the hierarchy of a health system or (b) that the actors at the top of the hierarchy can act successfully in isolation or in their own interest. The health system is not a closed system and therefore actors, such as international technology firms, can influence public debate in the public sphere and shape public trust in the health system. A study from Spain about social networks’ engagement during the COVID-19 pandemic confirms this open sphere and shows how the communication paradigm changed, giving social media a much more prominent place than before.^79^ As the health system is an open system and many understand the government to be ultimately responsible for the health system, even if it is not publicly owned, the health system is susceptible to spill-over effects of low levels of public trust in the government in other spheres.^41^ To counteract these spill-over effects, collaborative approaches are preferable. If health policymakers want to remain influential in the debate, they need to engage with a range of actors at an early stage, engage strongly in public discourse and not withdraw from public debate.

To understand changing levels of public trust and to use public trust as an outcome measure for health system performance evaluation, levels of public trust need to be monitored. As a result of such monitoring, policymakers and thus policies need to respond to changing levels of public trust. This response could involve professional training and codes of conduct, implementation of active regulatory systems^19^ or conscientious policy planning. Consequently, an open debate is necessary to understand and learn from adverse incidents affecting trust as soon as they occur rather than covering them up.

### Keep the public safe

A perception of safety and security is necessary for all parts of the health system to operate well and is by no means exclusive to the management of personal data. Security and safety in the health system are important means to build public trust and other forms of trust in health care.^12,80–82^ Here, safety could refer to the prevention of harm and medical errors. Inkster and colleagues propose five areas of action to ensure safety, long-term sustainability and availability of digital health management during and beyond the COVID-19 pandemic: ‘ (1) due diligence: remove harmful health apps from app stores; (2) data insights: use relevant health data insights from high-quality digital tools to inform the greater response to COVID-19; (3) freely available resources: make high-quality digital health tools available without charge, where possible and for as long as possible, especially to those who are most vulnerable; (4) digital transitioning: transform conventional offline mental health services to make them digitally available; and (5) population self-management: encourage governments and insurers to work with developers to look at how digital health management could be subsidized or funded’.^83^

### Offer autonomy to the public

Personal autonomy, for example, personal control over private information or the free choice to consent to research and health care interventions, is one of the core principles inherent in any process of establishing trust. However, to make choices within the context of health care is difficult for lay people and often ‘real’ choices are not provided by health professionals.^84^ For example, the quality of the alternatives may be far from equal, such that no genuine choice is being offered. This implies that the choices offered need to be of equal potential value and the people who want to make choices need to learn the skills to make the choices. This is shown in the ethical debate about whether the use of COVID-19 tracing apps should be mandatory or not and the extent to which compulsion harms or grows public trust. For example, in India, app use was mandatory for citizens ‘living in virus-containment zones and for all government and private sector employees. Apps in Argentina and the United Kingdom asked users to self-report their symptoms, whereas the Norwegian app relied on the user having a formal diagnostic test’.^85^ Basu argues in the Indian context that ‘the demonstration of trust through an emphasis on transparency, predefined limits to data collection, usage and data destruction timelines related to a contact tracing app should be adequate for instilling confidence in the reasonable individual even in the absence of voluntariness.’.^86^ Morley and colleagues argue that it is less ethical to implement a mandatory app in contrast to an optional download and installation.^85^ From a trust building and trust theory perspective, it is difficult to argue that autonomy to place or withdraw trust should be abandoned.^87^ Yet, there are situations where restricting personal freedom for the anticipated good of the wider public is justified. In such cases, a trusted governance framework needs to be in place for safe data use and protection of privacy.

### Plan for diverse trust relationships

Public trust includes several types of trust, namely: self-confidence (i.e. trust in oneself); interpersonal trust; and individual trust in the health system. Self-confidence is important for members of the public to be able to engage in any other trusting relationships. Low levels of self-confidence hinder the establishment of trust for all parties in the trusting relationship.^88^ As the public consists of private people engaging with health system representatives, interpersonal trust, as well as individual trust in the wider health system, are both important. Gasser and colleagues show that different technologies to fight COVID-19 (e.g. apps, symptom checkers for self-diagnosis or quarantine compliance) involve different actors (government, academics and private citizens) which we know from trust research enjoy different levels of trust.^16,32,89^ This implies that policies aimed at building public trust should consider that not all actors are equally trusted within the health system. Yet, to make an effective health policy against COVID-19, trust in a wide range of actors is needed.^2^ Policymakers need to examine the actors and their trust relationships central to each policy and design their trust-building strategies accordingly. It is unlikely that a ‘one-size-fits-all’ approach will succeed. From a health policy communication viewpoint, it will be advantageous to consider which actor is the most trusted and therefore might be most suitable to be the main communication source as a representative of the health system activity in the eyes of the public.

### Recognise that trust is shaped by both emotion and rational thought

Modern trust theory tends to see the process of placing one's trust in another person or institution as primarily a calculated, conscious decision. Despite this widely accepted understanding, there exists a different body of literature that identifies trust as the result, in part at least, of emotions and, therefore, as much motivated by ‘gut feeling’ as calculation.^90,91^ Min and colleagues, show in the context of COVID-19, that the ‘relationship between government trust and preventive behaviours was moderated by negative emotion’^92^ such as sadness, anger, fear or shocks. As negative emotions can be associated with loss of personal control,^92^ health policymakers need to provide future clarity and restore personal control, which relates to autonomy. Other research shows that anger and fear, in particular, are widely experienced emotions arising from the COVID-19 pandemic.^93^ To build public trust, such emotions need to be addressed by health system actors and they need to show how they intend to mitigate such public emotions. Also, health system actors should do their best to increase future certainty. Logically, it is impossible to foresee the future with exactitude. However, one can explicitly discuss the degree of uncertainty and provide possible future scenarios about how the health system response might evolve. Also, one can implement evaluation cycles as well as checkpoints in the policy process to give the public more confidence in the policy process. As a form of rational scepticism, deliberately placed checking (e.g. at quality control points) is likely to foster overall trust. Another example is members of staff in a policy team monitoring each other's performance.^94^ It is important to strike a fair balance between checking and trusting. If checking is to have value, the person checking must either be able to act fairly directly to ‘fix’ the problem or have the support of people/agencies that can act on his/her behalf. There is little value in checking by a regulator if all that happens is the identification of a problem or articulation of a criticism without it being clear how improvement can be made.

### Represent the public interest

The public expects that the government will represent the public interest and organise a health system that acts on their behalf. The relationship between the government and the public fails as soon as the providers of care are perceived as acting in their own interest and/or acting at the expense of the public interest. This means more specifically that the health care system needs always to focus on its impact on patients and the public. It is a necessity that health system actors act in the public interest by following good governance principles such as the United Kingdom's Nolan Principles of selflessness, integrity, objectivity, accountability, openness, honesty and leadership.^95^ If policymakers or politicians are perceived by the public as an egocentric elite, the public will be less trusting.^89^ The COVID-19 pandemic shows how governments can lose trust due to cronyism and blundering which are certainly not new phenomena.^96–98^

Respect between the trusting parties is essential. At the professional level, the maintenance of professional conduct with a focus on respectful behaviour towards patients and the public is key. This manifests itself in careful handling and management of personal data, for example, not losing data because of indifference, but also not getting so tied up with paternalistic regulation that personal data cannot be used effectively and efficiently for analysis and research designed to improve services. Equally, patients and healthy people need to interact with health care actors such as medical professionals or politicians in a respectful and honest way. It is unlikely that outbursts of rage, disrespectful comments or discriminatory comments as frequently seen on social media platforms will foster trust.^99,100^ Manners are key and it is commonly understood that respectful interaction strengthens relationships and fosters trust. It is unlikely that a person would trust someone s/he disrespects.

A breach of privacy undermines public trust. Privacy is likely to be maintained by professional behaviour as well as attempts to guarantee anonymity. Policymakers need to be explicit with the public about the limits of anonymity in relation to personal information.^24^ Privacy is maintained by not leaking, losing or distributing private information inappropriately, regardless of the degree of anonymity. In practice, this implies that the focus with respect to keeping data private must be more on the norms, expectations and behaviour of staff working with the data and less on external regulation. Specifically, privacy concerns are a major obstacle to the implementation and public acceptance of health apps in general as well as COVID-19 tracing apps and therefore need to be addressed appropriately.^31,64^ Advocates for the use of health apps and data use more broadly need to ‘a) openly discuss and explain the benefits and risks concerning identification; b) empower citizens by engaging them in decisions about health data use; and, c) demonstrate how privacy can be protected despite not being able to provide full anonymity.’^24^

### Work towards realising a net benefit for the health system and the public

The public trusts the health system in anticipation of a net benefit that comprises of a benefit to others, personal benefit, financial benefit and health system benefit. Altruistically motivated participation in health care research should lead to the benefit of others and future generations. The COVID-19 pandemic shows that the public is prepared to be altruistic.^101^ Consistent with this, data access within the health system, as, for example, access to donated data, should be organised in such a way that any benefit to private companies from the use of data is exceeded by the benefits obtained for the public system and thence the public.^102^ Public trust is particularly likely to be undermined if private companies can use the public health system and its data for their own profit without this also financially benefiting the public realm. Aside from a financial benefit, health system benefit relates to advances in science as well as improved quality of health care due to research. Quality of services, in turn, contributes to trust in the health system.^81,82,103,104^ Examples of advances in science can range from improved surgical techniques and shortened hospital stays to personalised medicine.

To be able to be trusted by the public to achieve this net benefit, the health system as a whole and its actors must have a recognisable potential to make decisions that produce net benefits for different groups in the population. As Gasser and colleagues write in relation to COVID-19 ‘Underpinning all scientific, ethical and legal challenges of pandemic management is the question of public benefit’.^16^ At the professional level, the ability to keep up with cutting-edge knowledge are important. At the governmental level, the government should act in a democratic way, research projects should be well designed and there should be a central, overriding public good objective when integrating private companies into publicly financed health services.

## Conclusion

Informed by a new conceptual framework of public trust in the health system, we derived guiding principles designed to foster public trust in data use within the health system, using examples drawn from the COVID-19 pandemic. It is essential to uphold public discourse to build and maintain public trust, and not to assume that public trust can be built by one-off action. Public trust is fragile and valuable. To be maintained, it needs constant communication and effort from all parties engaged in public discourse relating to the health system. As the debate on issues of trust in the public sphere is open to all members of the public and actors in society, the public trust that results from such debate legitimises state action and governance. If public trust in the health system (or parts of the health system), including the government, is missing, the health system is at risk of failing, as it risks being increasingly perceived by the public as lacking legitimacy. Subsequently, the public is more likely to withdraw its mandate.

The set of guiding principles now needs to be used to inform policymaking in health care so that they can be tested in practice. Also, the guiding principles could be used by researchers to analyse the performance of different governments during the pandemic to see if those governments that operated in greater conformity with the guiding principles performed better especially in the long run.

## Supplemental Material

sj-xlsx-1-dhj-10.1177_20552076221111947 - Supplemental material for Evidence-based guiding principles to build public trust in personal data use in health systems Click here for additional data file. Supplemental material, sj-xlsx-1-dhj-10.1177_20552076221111947 for Evidence-based guiding principles to build public trust in personal data use in health systems by Felix Gille, Sarah Smith and Nicholas Mays in Digital Health
